# Resting-state fMRI in women with functional constipation with or without stress urinary incontinence

**DOI:** 10.3389/fneur.2026.1744318

**Published:** 2026-04-13

**Authors:** Yule Xie, Wenjie Dang, Xuzhao Li, Kai Ai, Hong Zhang, Min Hu, Rui Zhao, Feng He, Rongrong Zhu

**Affiliations:** 1 Medical Imaging Center, People’s Hospital of Ningxia Hui Autonomous Region, Ningxia Medical University, Yinchuan, China; 2 General Surgery Center, People’s Hospital of Ningxia Hui Autonomous Region, Ningxia Medical University, Yinchuan, China; 3 Department of Clinical and Technical Support, Philips, Xi’an, China; 4 Department of Neonatology, Peking University First Hospital Ningxia Women and Children’s Hospital, Yinchuan, China

**Keywords:** amplitude of low-frequency fluctuation, functional connectivity, functional constipation, functional magnetic resonance imaging, stress urinary incontinence

## Abstract

**Background:**

No prior research has investigated whether abnormalities exist in the neural regulation mechanisms of patients with comorbid Functional Constipation (FCon) and Stress Urinary Incontinence (SUI). This study employed neuroimaging to examine differences in brain activity and functional connectivity between female FCon patients with and without SUI.

**Materials and methods:**

Resting-state fMRI data were prospectively obtained from 34 female patients diagnosed with FCon comorbid with SUI (FCon-SUI), 24 female patients with FC without SUI (FCon-NSUI), and 29 Healthy Controls (HC). The study compared the Amplitude of Low-Frequency Fluctuation (ALFF) among the three groups to identify regions manifesting abnormal local spontaneous neural activity. Regions demonstrating significant ALFF variances were subsequently utilized as seeds for seed-based functional connectivity (FC) analysis. Additionally, correlations between brain functional irregularities and clinical symptoms were examined.

**Results:**

FCon-SUI and FCon-NSUI patients exhibited aberrant ALFF and FC values across various brain regions. Specifically, the FCon-SUI cohort demonstrated elevated ALFF in the right supplementary motor area (SMA) and the right middle frontal gyrus (MFG), as well as reduced ALFF in the left inferior temporal gyrus (ITG) relative to the FCon-NSUI group. In FCon-SUI, right SMA ALFF was positively correlated with KESS scores.

**Conclusion:**

Our findings highlight the unique neural activity characteristics of FCon-SUI, and provide valuable insights for monitoring brain changes in FCon-SUI patients and identifying potential therapeutic targets.

## 1 Introduction

Female Pelvic Floor Dysfunction (FPFD) (1) is a common condition in middle-aged and elderly women, characterized by defects, impairments, or functional abnormalities in the pelvic floor support structures. It affects around 40% of women and manifests through symptoms such as functional constipation (FCon), stress urinary incontinence (SUI), pelvic organ prolapse, and sexual dysfunction (2). The symptoms of FPFD can be subtle and overlapping; about 40% of women may experience a single symptom in their lifetime, while 17% may have multiple symptoms (3). Additionally, researchers have noted a frequent co-occurrence of FCon and SUI in FPFD patients (4).

FCon is a prevalent functional gastrointestinal disorder (FGID) characterized by infrequent bowel movements, hard stools, and straining during defecation (5), affecting approximately 15% of the global population (6). The pathophysiology of FCon is intricate, involving the autonomic and somatic nervous systems, anal sphincters, and pelvic floor muscles (7). Some scholars suggest that the initiation of FCon is closely linked to dysfunction in the brain-gut axis (8). Several studies have indicated a potential correlation between constipation and urinary incontinence (9, 10). The repetitive elevation of intra-abdominal pressure due to chronic bowel irregularities may contribute to the onset of urinary incontinence by compromising the support structures of the pelvic floor and disrupting pelvic autonomic nerve function.

Urinary incontinence, characterized by the involuntary release of urine, is commonly categorized into subtypes such as stress urinary incontinence (SUI), urgency urinary incontinence (UUI), and overflow urinary incontinence (OUI), with SUI being the most prevalent (11). In China, the prevalence of SUI among adult women is reported to be as high as 18.9%, significantly impacting physical and mental well-being and imposing a substantial healthcare burden (12). The primary pathogenesis of SUI is associated with anatomical changes influenced by risk factors like age, childbirth, obesity, and constipation (13), including intrinsic urethral characteristics, periurethral supporting structures, and pelvic nerve integrity (14). However, emerging research highlights the role of central nervous system dysfunction in urinary incontinence, particularly for UUI and overactive bladder (OAB). For example, studies using resting-state functional magnetic resonance imaging (rs-fcMRI) have demonstrated abnormal functional connectivity in women with UUI, suggesting that rs-fcMRI may predict individual disease presence and severity (15). Additionally, abnormal resting-state networks have been identified in females with OAB, further implicating disrupted brain networks in bladder control dysfunction (16). These findings support the theoretical model of brain-bladder control, which proposes that dysfunction in specific brain regions contributes to voiding abnormalities (17).

In recent years, various neuroimaging techniques have been utilized to investigate the neural mechanisms underlying the development of FCon or SUI. Studies have demonstrated notable variances in baseline brain activity in specific regions among FCon patients compared to healthy individuals. These regions are linked to emotional regulation (specifically the dorsal anterior cingulate cortex-dACC and hippocampus-HIPP), somatic and sensory processing, and motor control (specifically the supplementary motor area-SMA) (18). Task-specific fMRI studies have further outlined altered brain responses to external stimuli in individuals with urinary incontinence (15). Nevertheless, the distinct neuroregulatory features in patients with concurrent FCon and SUI have not been thoroughly investigated. Studies have identified that motor neurons controlling the rectum and bladder originate from pelvic parasympathetic ganglia situated within sacral segments S2 and S4, with overlapping pudendal nerve pathways supplying the external anal and urethral sphincters (9). This anatomical overlap implies potential shared central nervous system regulatory targets for both conditions.

Resting-state functional magnetic resonance imaging (rs-fMRI) was employed to capture intrinsic brain dynamics by recording spontaneous blood oxygen level-dependent (BOLD) signal fluctuations, without necessitating subject engagement in specific tasks (19). This imaging modality has gained considerable prominence in modern neuroscience research, offering more comprehensive exploration of abnormal neural processes compared to task-activated fMRI approaches. Key analytical methods include assessing the amplitude of low-frequency fluctuations (ALFF) and functional connectivity (FC), both crucial parameters in neuroimaging studies. ALFF measures the intensity of spontaneous low-frequency BOLD oscillations, reflecting regional neuronal activity levels (20–22). Conversely, FC analysis investigates temporal correlations among different brain regions, unveiling patterns of interregional neural synchronization. Scholarly inquiries often integrate ALFF and FC metrics to comprehensively delineate cerebral functional changes.

In this study, we utilized the ALFF technique to examine changes in regional neural activity across three cohorts: FCon with SUI (FCon-SUI), FCon without SUI (FCon-NSUI), and Healthy Controls (HC). Following this, a seed-based whole-brain analysis of resting-state functional connectivity (rsFC) was conducted. Discrepancies in neural activation and functional connectivity are anticipated to offer novel perspectives for identifying prospective therapeutic interventions.

## 2 Materials and methods

### 2.1 Participants

This study conducted a prospective recruitment of female patients with FCon from the Department of Gastrointestinal Surgery at Ningxia Hui Autonomous Region People’s Hospital between August 2023 and June 2025. All participants were right-handed females aged between 40 and 70 years (23). Patients were categorized into two groups based on the presence of SUI: FCon-SUI and FCon-NSUI. The cohort comprised 34 FCon-SUI patients, 24 FCon-NSUI patients, and 29 HC were age-matched volunteers without gastrointestinal or urinary symptoms, recruited from the same hospital. Written informed consent was obtained from all participants, and ethical approval for the Ethics Committee of People’s Hospital of Ningxia Hui Autonomous Region (reference: 2025-NZR-053).

#### 2.1.1 Patient inclusion criteria

FCon diagnosis was confirmed by gastroenterologists certified in FGIDs using Rome IV criteria, requiring: (1) At least 25% of the time defecation is laborious; (2) At least 25% of the time stool characteristics are lumpy or hard; (3) At least 25% of the time there is a feeling of incomplete defecation; (4) At least 25% of the time defecation has anorectal obstruction or obstruction; (5) At least 25% of the time defecation requires manual assistance; (6) Spontaneous defecation <3 times per week; (7) There is rarely loose stool without the use of laxatives. Any of the above (except item 2) plus type 1 or type 2 of Bristol stool typing with symptoms present for at least 6 months (24).

#### 2.1.2 SUI diagnosis required

(1) Documented history of urine leakage during stress maneuvers (coughing/Valsalva) (25); (2) The patients got MR defecography for assessment of SUI (26); (3) The diagnosis of SUI is supported by urodynamic test results. Additionally, inclusion criteria encompassed the patient’s willingness to participate in fMRI examinations and their capacity to comprehend, consent to, and adhere to the study protocols.

#### 2.1.3 Exclusion criteria included

(1) Constipation resulting from organic lesions or medication. (2) Pelvic masses causing significant compression as determined by physical examination or MRI. (3) History of pelvic surgery, including prior pelvic floor repair. (4) Concurrent urinary tract infection or gynecological inflammatory conditions. (5) History of severe neuropsychiatric disorders (e.g., schizophrenia, bipolar disorder, organic mental disorders affecting brain function and pelvic floor regulation). (6) Ineligibility for MRI or inability to cooperate during the MRI procedure.

### 2.2 Questionnaire assessments

General clinical data and constipation symptom evaluation encompassed age, disease duration, and body mass index (BMI) for all participant groups. Constipation symptom severity in the two FCon cohorts was assessed utilizing the Knowles-Eccersley-Scott Symptom Questionnaire (KESS) (27).

### 2.3 fMRI data acquisition protocol

All subjects emptied their bladders prior to fMRI scanning, and changed into standard scrubs with all ferromagnetic objects removed. Participants were positioned supine and fitted with noise-cancelling headphones and a foam headrest. They were instructed to keep their eyes closed, maintain a natural breathing rhythm, avoid abnormal spontaneous brain region activation induced by emotional stress. Imaging was conducted using a Philips Ingenia CX 3.0 T MRI system with a 32-channel head coil. Functional imaging employed a BOLD contrast gradient-echo echo-planar imaging (EPI) sequence with the following parameters: 51 contiguous axial slices, slice thickness = 5.00 mm, repetition time (TR) = 2000 ms, echo time (TE) = 35 ms, field of view (FOV) = 219 × 219 mm^2^, acquisition matrix = 88 × 88, flip angle = 65°, and total scan duration = 420 s. Three-dimensional structural images were obtained using a T1-weighted magnetization-prepared rapid gradient-echo (MPRAGE) sequence with the following parameters: 280 contiguous sagittal slices, no gap, TR = 7.6 ms, TE = 3.6 ms, FOV = 250 × 250 mm^2^, matrix = 228 × 227, flip angle = 8°.

#### 2.3.1 Resting-state data processing

Preprocessing was performed in MATLAB R2023b using SPM12 and DPABI with the following pipeline: DICOM-to-NIFTI format conversion. Exclusion of initial 10 volumes for magnetic stabilization. Slice timing correction. Realignment with motion scrubbing (exclusion threshold: >2 mm translation or >2°rotation). Spatial normalization to MNI space (3 × 3 × 3 mm^3^ voxels). Gaussian spatial smoothing (6 mm FWHM). Temporal filtering (0.01–0.1 Hz bandpass). Regression of nuisance covariates (Friston 24-parameter motion, WM/CSF signals) and linear detrending.

#### 2.3.2 ALFF calculation

To measure spontaneous neural activity strength, the ALFF approach was applied. This involved filtering the preprocessed time series with a band-pass filter (0.01–0.1 Hz) to attenuate low-frequency drift and physiological noise. Next, spectral power was derived in the frequency domain via the Fast Fourier Transform (FFT). After applying a square root transformation to the power values, the mean amplitude across the target frequency band (0.01–0.1 Hz) was determined for every voxel. Finally, each voxel’s ALFF value was standardized by dividing it by the mean ALFF value of the entire brain, and standardized ALFF maps were subsequently generated.

#### 2.3.3 Seed-based FC analysis

RsFC analysis was conducted by employing seed-based whole-brain FC analysis with regions showing notable group differences as identified in the ALFF analysis serving as seed regions. Initially, the normalized time-series signals were extracted from each region of interest (ROI). Subsequently, Pearson correlation coefficients were utilized to evaluate the temporal synchrony between each seed region and all other voxels throughout the entire brain, resulting in functional connectivity strength maps.

## 3 Statistical analysis

Statistical analyses were performed using SPSS 27.0 (IBM) and SPM12. Age and BMI comparisons across FCon-SUI, FCon-NSUI, and HC cohorts employed one-way ANOVA. Group disparities in illness duration and clinical indices were assessed via independent samples t-tests, with statistical significance defined as *p* < 0.05.

For rs-fMRI second-level analysis, SPM12 software, whole-brain ALFF values and seed-to-voxel rsFC strengths were compared using one-way ANCOVA with age and BMI as covariates. Multiple comparisons correction applied voxel-wise false discovery rate (FDR) at cluster-level *p* < 0.05 (voxel height threshold *p* < 0.001). Regions demonstrating significant ALFF/ALFF-based rsFC alterations (*p* < 0.05) underwent post-hoc Bonferroni-corrected ANOVA. Pearson correlations quantified associations between clinical variables and aberrant ALFF/FC values at *p* < 0.05 significance threshold.

## 4 Results

### 4.1 Demographic and clinical characteristics

This investigation included 34 participants diagnosed with FCon-SUI, 24 individuals with FCon-NSUI, and 29 HC displays the demographic and clinical profiles (Table 1).

**Table 1:** Demographic and clinical data of FCon-SUI, FCon-NSUI, and HCs groups.

Variables	FCon-SUI (*N* = 34)	FCon-NSUI (*N* = 24)	HC (*N* = 29)	*p* value
Age (years)	59.47 ± 8.47	53.38 ± 11.44	52.52 ± 8.12	0.007^a^
BMI	25.19 ± 2.52	22.56 ± 2.28	22.36 ± 3.42	<0.001^a^
Illness duration (years)	9.56 ± 6.81	8.33 ± 5.26		0.46^b^
KESS	27.32 ± 4.42	23.17 ± 5.25		0.002^b^

FCon-SUI: FCon with Stress Urinary Incontinence; FCon-NSUI: FCon but without SUI; HC: Healthy Controls; BMI: body mass index; KESS: Knowles-Eccersley-Scott Symptom Questionnaire.

a *p*-values obtained by ANOVA model.

b *p*-value obtained by Two-sample t-tests.

Significant variations were noted among the three cohorts concerning age and body mass index BMI (*p* < 0.05). While there was no notable distinction in disease duration between the two patient groups (*p* > 0.05), a statistically significant variance was observed in the KESS score (*p* < 0.005).

### 4.2 ALFF results

**Figure 1:**
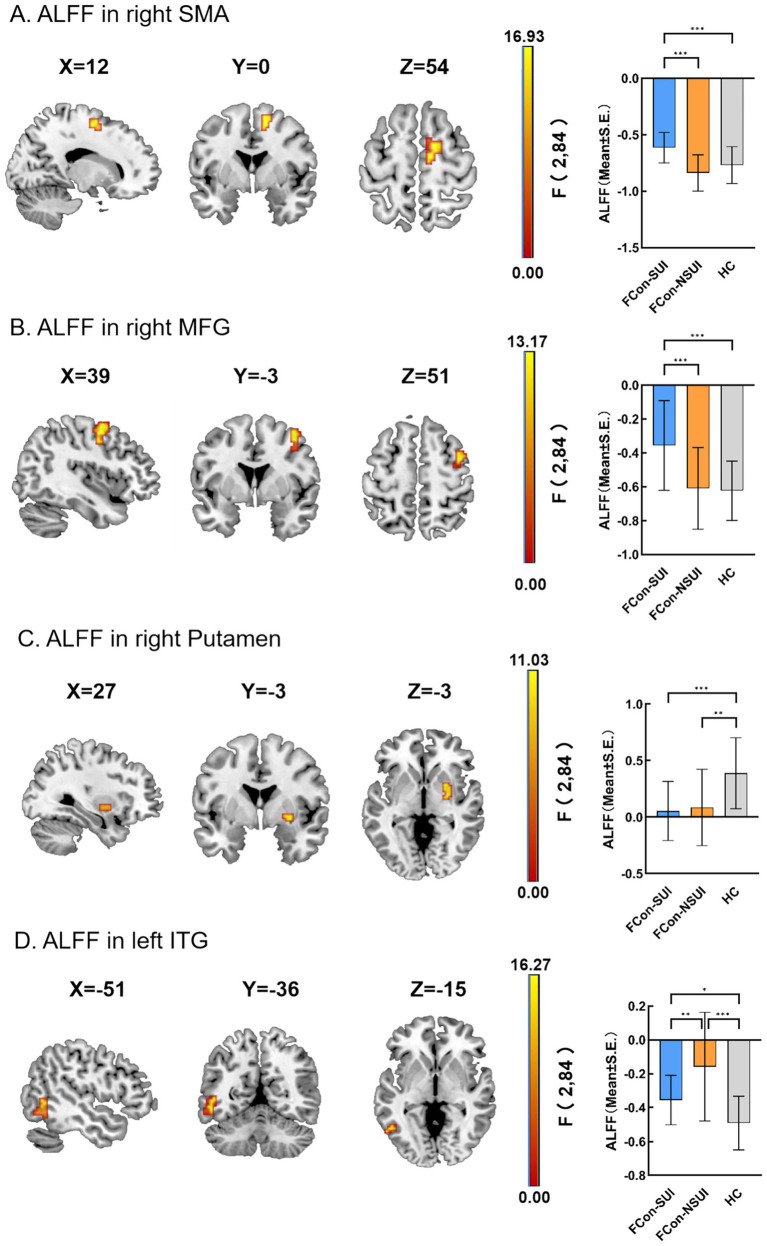
Regional ALFF differences. Left panels: statistical maps (sagittal/coronal/axial) highlighting clusters with significant differences. Right panels: group-level ALFF values in in right SMA (right supplementary motor area) **(A)**, right MFG (right middle frontal gyrus) **(B)**, right putamen **(C)**, and left ITG (left inferior temporal gyrus) **(D)**.

ALFF comparisons across FCon-SUI, FCon-NSUI, and HC cohorts are summarized in Tables 2, 3 and Figure 1. Significant differences were observed among the three cohorts in specific brain regions, including the right supplementary motor area (SMA), right middle frontal gyrus (MFG), right putamen, and left inferior temporal gyrus (ITG) (FDR-corrected; voxel-level *p* < 0.001, cluster-level *p* < 0.05). Subsequent analysis indicated that the FCon-SUI group exhibited increased ALFF values in right SMA, right MFG, and left ITG, while decreased ALFF values were noted in right Putamen compared to the HC group. Comparatively, the FCon-NSUI group displayed increased ALFF in left ITG and decreased ALFF in right Putamen relative to the HC group. Furthermore, when contrasting the FCon-NSUI group with the FCon-SUI group, heightened ALFF values were observed in right SMA and right MFG, whereas decreased ALFF values were detected in left ITG.

**Table 2:** Brain regions with significantly altered ALFF and corresponding statistical parameters.

Altered region^*^	MNI coordinates (X, Y, Z)			Cluster size	F value	Partial η^2^
Right SMA	12	0	54	43	10.18	0.187
Right MFG	39	−3	51	37	9.57	0.197
Right putamen	27	−3	−3	25	11.43	0.216
Left ITG	−51	−36	−15	42	12.34	0.229

MNI: Montreal Neurologic Institute; SMA: Supplementary Motor Area; MFG: Middle Frontal Gyrus; ITG: inferior temporal gyrus. *Anatomical labeling of brain regions was based on the Automated Anatomical Labeling 3 (AAL3) atlas.

**Table 3:** Between-group comparisons of ALFF values: T-statistics and Cohen’s d.

Cluster location	T-value^a^	Cohen’s d
FCon-SUI vs. HC		
Right SMA	4.03	0.71
Right MFG	4.57	0.81
Left ITG	2.53	0.45
Right putamen	−4.38	0.78
FCon-NSUI vs. HC		
Left ITG	5.70	1.06
Right putamen	−3.64	0.68
FCon-SUI vs. FCon-NSUI		
Right SMA	5.54	0.94
Right MFG	4.10	0.70
Left ITG	−3.49	0.60

a Positive and negative T-values correspond to increased and decreased ALFF, respectively.

### 4.3 ALFF-based rsFC results

**Figure 2:**
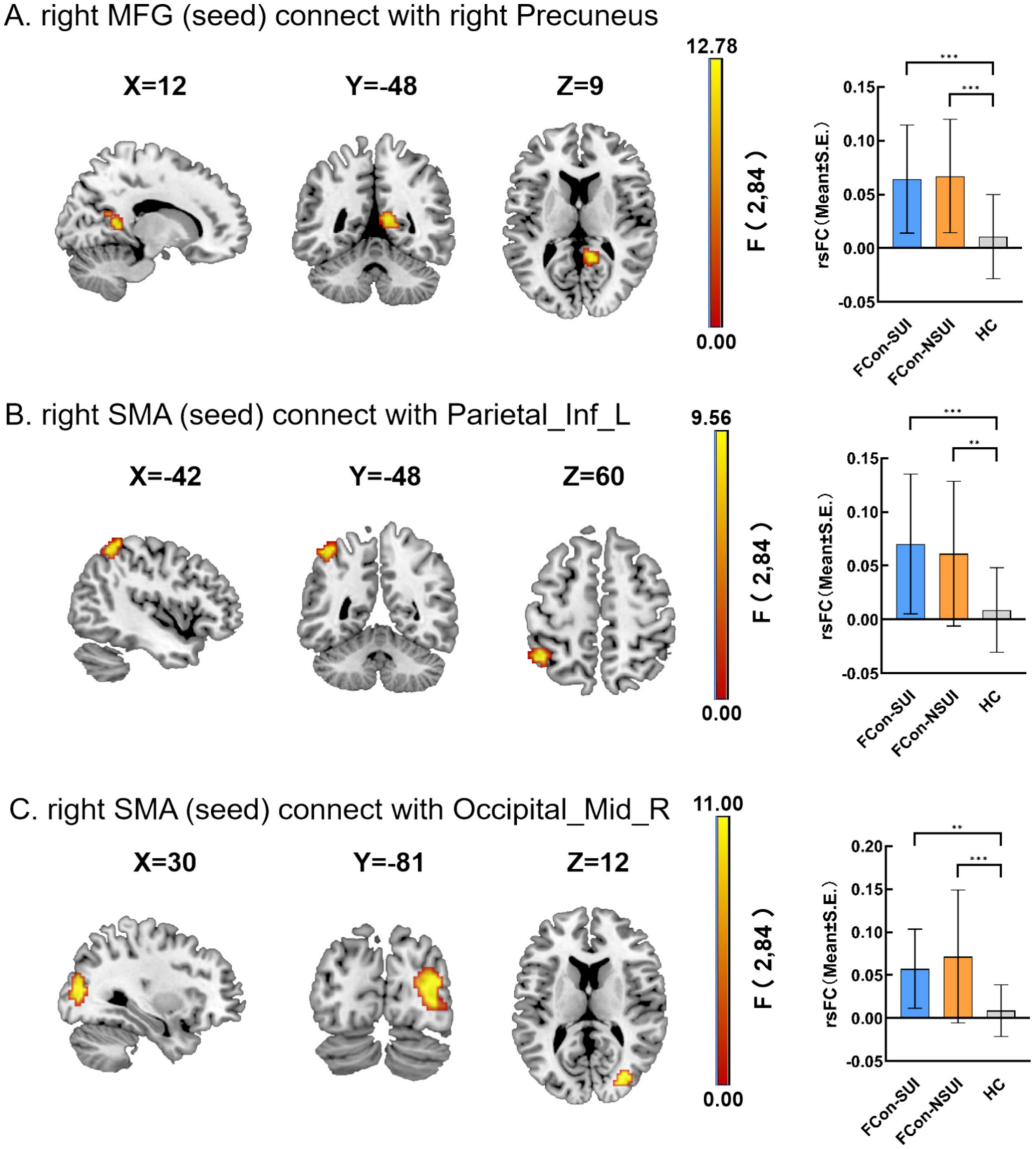
Seed-to-voxel rsFC mappings. The comparison of rsFC between the right MFG (seed) and right precuneus **(A)**, the comparison of rsFC between the right SMA (seed) and Parietal_Inf_L **(B)**, the comparison of rsFC between the right SMA (seed) and Occipital_Mid_R **(C)**. In panels **(A–C)**, the left part shows the statistical maps (sagittal/coronal/axial orientations), and the right part is a group-level rsFC strength in significant clusters.

As shown in Tables 4, 5 and Figure 2 (FDR-corrected, the voxel threshold was *p* < 0.001, the cluster threshold was *p* < 0.05), a seed-to-voxel rsFC analysis was conducted using the ALFF analysis results as seeds.

**Table 4:** Seed-to-voxel FC analysis results.

Seed	Connected region	MNI coordinates (X, Y, Z)			Cluster size	*F* value	Partial *η^2^*
Right MFG	Right precuneus	12	−48	9	47	14.56	0.72
Right SMA	Parietal_Inf_L	−42	−48	60	34	12.89	0.67
	Occipital_Mid_R	30	−81	12	74	18.54	0.81

MNI: Montreal Neurologic Institute; SMA: Supplementary Motor Area; MFG: Middle Frontal Gyrus; Parietal_Inf_L: left inferior parietal lobule; Occipital_Mid_R: right middle occipital gyrus.

**Table 5:** Between-group comparisons of functional connectivity strength: T-statistics and Cohen’s d.

luster location	T-value^a^	Cohen’s *d*
FCon-SUI vs. HC		
Right precuneus	4.15	0.74
Parietal_Inf_L	4.45	0.79
Occipital_Mid_R	3.67	0.65
FCon-NSUI vs. HC		
Right precuneus	3.25	0.60
Parietal_Inf_L	4.29	0.80
Occipital_Mid_R	4.35	0.81

a Positive and negative T-values correspond to increased and decreased ALFF, respectively.

As shown in Figure 2A, employing the right MFG as the seed region, both FCon-NSUI and FCon-SUI patient cohorts displayed significantly heightened rsFC between right MFG (seed) and the right precuneus in comparison to the HC group. Additionally, as displayed in Figures 2B,C, using right SMA as the seed region, both FCon-NSUI and FCon-SUI patient groups exhibited notably increased rsFC between right SMA (seed) and the left inferior parietal lobule (Parietal_Inf_L) as well as between right SMA (seed) and the right middle occipital gyrus (Occipital_Mid_R) relative to the HC group. No significant differences in rsFC were observed between the FCon-NSUI and FCon-SUI patient groups. Furthermore, no significant differences in rsFC were detected when the left ITG was used as the seed region for rsFC analysis.

### 4.4 Correlation of clinical data with ALFF and rsFC

**Figure 3:**
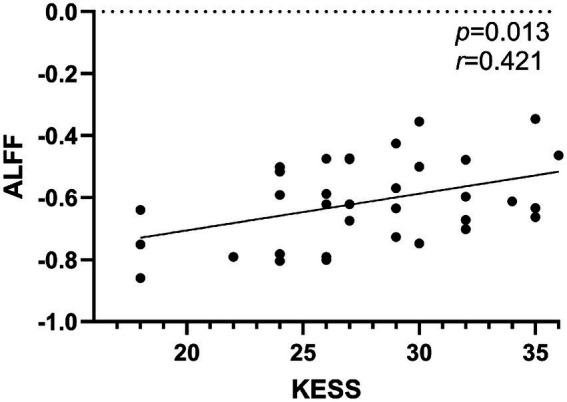
Significant correlations (*p* < 0.05) between clinical indices and aberrant ALFF values are demonstrated in scatter plots across the cohort.

As shown in Figure 3, Pearson’s correlation coefficients unveiled a significant positive association between ALFF values in right SMA and KESS scores in FCon-SUI patients (*r* = 0.421, *p* = 0.0131, 95% *CI*:0.105 ~ 0.737). In contrast, no significant correlations were found between KESS scores and ALFF values in either the right MFG or the left ITG. Additionally, No significant correlations were identified between rsFC values and clinical parameters.

## 5 Discussion

This study employed integrated ALFF and seed-based rsFC analyses to investigate neurofunctional alterations at regional and network levels in three cohorts: FCon-SUI, FCon-NSUI and HC. The multimodal approach identified distinct neural signatures associated with clinical symptoms, revealing significant intergroup differences that correlated with symptom severity.

The SMA, a fundamental component of the sensorimotor network (SMN), is essential for coordinating complex body movements and integrating sensory feedback to enhance motor control (28). It also regulates pelvic floor muscle movements by modulating voluntary contractions and processing sensory signals from the body (29). In this study, the ALFF values in the right SMA were significantly higher in patients with FCon-SUI compared to HC and patients with FCon-NSUI. This finding indicates increased resting neural activity in the right SMA, which is associated with the physical abnormalities observed in chronic pelvic floor muscle contraction disorders (30). Such abnormal overactivity disrupts the brain’s precise control of the anal and urethral sphincters, potentially contributing to the bowel and bladder dysfunction experienced by these patients. Furthermore, elevated right SMA ALFF values were significantly correlated with higher KESS ratings, demonstrating that greater neural activity abnormalities in this region are linked to more severe constipation. Collectively, these results suggest that the right SMA may serve as a potential target for central neuromodulation therapies, such as transcranial magnetic stimulation (TMS) (31, 32), in individuals with FCon-SUI.

The MFG, a crucial subregion of the dorsolateral prefrontal cortex (DLPFC), plays a vital role in advanced cognitive regulation and the inhibition of unwanted visceral motor movements (33, 34). Prior research has identified structural alterations in the MFG of patients with functional constipation (35), and its compromised function is associated with the onset of urinary dysfunction (36). Patients with FCon-SUI exhibited significantly elevated ALFF values in the right MFG, indicating that this brain region modulates its neural activity in response to prolonged pathological conditions. This adaptive change likely means that the brain allocates additional cognitive resources to compensate for diminished voluntary control over pelvic floor muscles. The MFG regulates pelvic floor movements by suppressing sudden, abnormal defecatory or urinatory urges and initiating goal-directed motor actions (37, 38). Collectively, these findings indicate that the right MFG may serve as a significant neural biomarker for FCon-SUI and a promising primary target for cognitive neuromodulation interventions.

The putamen, the primary input nucleus of the basal ganglia, plays a crucial role in regulating voluntary body movements and integrating cortical–limbic signals to modulate visceral motor activity and sensory perceptions (39–41). It is also integral to the neural network that coordinates the functions of the anal and urethral sphincters (16, 18, 42). Both FCon-NSUI and FCon-SUI patients exhibited significantly lower ALFF values in the right putamen compared to healthy controls, suggesting a reduction in baseline neural activity in this region. This decrease is associated with the putamen’s impaired capacity to regulate pelvic floor muscle tone and inhibit abnormal urges for defecation or urination. No significant difference in right putamen ALFF values was observed between FCon-NSUI and FCon-SUI patients. This null finding may be partially attributed to the limited sample size, which hindered the detection of subtle statistical differences. Importantly, this result has mechanistic implications: abnormal neural activity in the putamen may be closely linked to the onset of functional constipation, and the comorbidity of stress urinary incontinence may also be related to dysfunction within this critical brain region.

The ITG plays a crucial role in integrating emotional and somatosensory information, maintaining strong functional connectivity with the limbic system (43). In this study, the ALFF values in the left ITG were significantly elevated in both the FCon-NSUI group and the FCon-SUI group compared to the HC. This finding indicates a potential link between chronic psychological distress associated with pelvic floor dysfunction (44) and heightened activity within the limbic system (45). Hyperactivation of the limbic system can exacerbate intestinal motility and sensory dysfunction through the hypothalamic–pituitary–adrenal (HPA) axis (46). This hyperactivation may further impair sensory and motor control of the bladder by regulating bidirectional information transmission along the brain-gut-bladder axis, which is a core regulatory pathway mediating coordinated pelvic floor organ dysfunction in functional intestinal and bladder disorders (47). This finding aligns with previous studies that have reported a clinical correlation between abdominal pain-related brain-gut interaction disorders and symptoms of overactive bladder (48). Notably, the ALFF values of the left ITG in the FCon-SUI group were lower than those in the FCon-NSUI group. This observation may indicate adaptive changes in the brain’s neural activity patterns in the context of comorbidity between these two conditions. Such manifestations might be linked to adjustments in the brain’s neural resource allocation strategies in response to intestinal-bladder comorbidity, suggesting that the mechanisms underlying ITG involvement in the bidirectional regulation of the brain-gut-bladder axis exhibit specific differences across distinct clinical phenotypes of pelvic floor dysfunction.

The rsFC analysis revealed abnormal connectivity patterns in two key neural systems in FCon-SUI patients: the default mode network-sensory integration circuit and the motor-visual compensatory pathway. These network-level connectivity abnormalities were consistent with regional ALFF alterations, collectively providing robust neuroimaging evidence for elucidating the pathological circuit characteristics of FCon-SUI. No significant differences were detected in rsFC analysis with the left ITG as the seed region. This result suggests that the regulatory role of the left ITG in functional constipation is primarily mediated by local neural activity changes, whereas its abnormal interregional connectivity may be too subtle to be identified by the rsFC methodologies and statistical thresholds used in the present study.

The precuneus is a fundamental component of the default mode network (DMN) and is involved in visceral autonomic neural responses as well as the integration of affective and cognitive processing (49). In this study, rsFC between the right MFG and the right precuneus was significantly elevated in both FCon-NSUI and FCon-SUI patients compared to the HC group. This adaptive neural alteration is associated with enhanced coupling between the prefrontal cortex and the DMN, and may be linked to abnormal visceral autonomic activity and impaired affective-cognitive processing in patients with functional constipation. These findings are inconsistent with previous studies that reported abnormal precuneus-thalamic connectivity in individuals with functional constipation and lower urinary tract dysfunction (50, 51). Such discrepancies may stem from differences in study cohort characteristics (e.g., disease subtypes, comorbidity profiles), neuroimaging analytical approaches, and the specific brain region connections focused on in each study.

The occipital lobe is the primary brain region subserving visual processing, and also participates in regulating spatial attention and integrating multisensory signals, including somatosensory and visuospatial information (52). In this study, rsFC between the right SMA and the right inferior occipital gyrus was significantly elevated in both FCon-NSUI and FCon-SUI patients compared to the HC group. This finding indicates that patients allocate neural resources to this circuit even in the absence of defecation, which is associated with the monitoring and suppression of pelvic floor-related urges and aversive sensations. It is also linked to altered connectivity patterns within resting-state brain networks. These results are consistent with prior research on FGIDs that reported enhanced SMA connectivity with other brain regions (53, 54), confirming that SMA dysfunction is intricately involved in the pathological processes underlying FGIDs. This enhanced connectivity may reflect a state of hypervigilance in the brain, triggered by persistent pelvic floor-visceral signals, resembling a pathological neural adaptation. Such a state may result in unnecessary sensory amplification and attentional fixation during non-defecation periods, potentially exacerbating symptom perception and disrupting normal defecation behavior. This observation aligns with the theoretical model of abnormal coupling between sensory and attentional networks in functional somatic symptom disorders (55). The elevated rsFC between the right SMA and the right inferior occipital gyrus offers a novel perspective for elucidating the central neural mechanisms underlying FCon and SUI. Furthermore, it underscores the intricate interrelation among motor control, sensory processing, and spatial attention regulation in these disorders.

The parietal cortex is a crucial brain region responsible for integrating high-order sensory signals and translating them into motor responses (56). It integrates diverse sensory inputs while subserving spatial perception and motor guidance (57). The precise regulation of pelvic floor organs relies heavily on the accurate transduction of sensory signals and multimodal integration. Clinical studies on chronic pelvic pain have shown that increased resting-state connectivity in the left frontoparietal cortex correlates with symptom alleviation in patients suffering from chronic pelvic pain syndrome of the urinary system (58). This finding suggests that neural circuits mediated by the parietal cortex can regulate pelvic floor function through compensatory enhancements in connectivity, thereby exerting significant regulatory effects in cases of pelvic floor dysfunction. When the regulatory functions of the pelvic floor are compromised, the parietal cortex compensatorily intensifies its role in processing pelvic visceral and somatosensory inputs, as well as in coordinating the regulation of the urethral and anal sphincters to offset deficits in core regulatory circuits. In this study, the rsFC between the right SMA and the right parietal cortex was significantly increased in both the FCon-NSUI and FCon-SUI patient groups compared to the HC group. This elevation primarily indicates the activation of a positive compensatory mechanism by the central nervous system, which aims to offset deficits in the core regulatory circuits of the pelvic floor. This mechanism seeks to enhance multisensory integration and motor planning, thereby striving to maintain the regulation of pelvic floor organs as close to normal as possible.

No significant differences in rsFC patterns were observed between the FCon-SUI and FCon-NSUI cohorts in this study. This null finding may be partially attributed to the limited sample size or variable severity of stress urinary incontinence among participants. Nonetheless, this result warrants further investigation to enable more nuanced interpretation. The pathogenesis of chronic constipation may be closely associated with abnormal functional connectivity within the core sensorimotor network identified in this study. Additionally, comorbid urinary incontinence symptoms may interact with functional impairments of the same central neural circuitry.

## 6 Limitations

First, this study employed a cross-sectional design, which identifies correlations between abnormal brain regional function, altered brain network connectivity, and the pathological state of FCon-SUI, but cannot establish the causal relationships or temporal sequence underlying these interactions. Additionally, the sample size is limited, and no refined stratified analysis was conducted based on the severity of SUI and constipation. Consequently, some potential intergroup differences may have been undetected, hindering the exploration of quantitative correlations between brain regional indices and clinical symptoms. Furthermore, the lack of standardized screening and quantitative assessment of anxiety and depressive symptoms in participants may introduce confounding biases into the analysis of neural activity and functional connectivity related to pelvic floor dysfunction. Future studies will address this deficiency by incorporating the Hamilton Depression Rating Scale (HAMD) and Hamilton Anxiety Rating Scale (HAMA) for rigorous subject screening, quantitative emotional analysis and stratified research, thereby eliminating psychological confounding effects and enhancing the reliability of study findings. Meanwhile, large-sample longitudinal cohort studies and interventional studies can be conducted to further verify the causal relationships between variables, thus providing a direction for the in-depth validation of the research conclusions.

## 7 Conclusion

This study compared regional neural activity and brain functional connectivity between FCon-SUI and FCon-NSUI patients using rs-fMRI combined with ALFF and rsFC analyses, with HC as the reference group. Significant baseline neural activity differences were identified in somatosensory and motor control-related brain regions across the three groups; additionally, both patient cohorts exhibited distinct functional connectivity abnormalities in core sensorimotor and cognitive regulatory networks relative to HC. Notably, aberrant neural activity and connectivity in the right SMA, right MFG, and left ITG were closely associated with the pathological features of FCon-SUI, suggesting these brain regions as potential targets for central neuromodulation therapies (e.g., TMS) in FCon-SUI patients. This study characterizes distinct neural activity profiles in FCon-SUI and FCon-NSUI patients, providing valuable insights for monitoring disease-specific brain functional alterations in FCon-SUI patients, identifying precise therapeutic targets, and formulating corresponding intervention strategies for this disorder.

## Data Availability

The original contributions presented in the study are included in the article/supplementary material, further inquiries can be directed to the corresponding author.
